# P-1105. Assessing Infection Prevention and Control Knowledge, Attitudes, and Practices among Healthcare Personnel in Skilled Nursing Facilities, in the City of Detroit, Michigan

**DOI:** 10.1093/ofid/ofaf695.1300

**Published:** 2026-01-11

**Authors:** Trini A Mathew, Jessica Lahiff, Samia Arshad, Matthew Seeger, Brenda M Brennan, Sara E McNamara, Paul E Kilgore

**Affiliations:** Beaumont Hospital – Royal Oak , Royal Oak, MI; Henry Ford Hospital, Royal Oak, Michigan; Wayne State University, Rochester Hills, Michigan; Wayne State University, Rochester Hills, Michigan; Michigan Department of Health and Human Services, Lansing, Michigan; State of Michigan, Lansing, Michigan; Eugene Applebaum College of Pharmacy and Health Sciences, Detroit, Michigan

## Abstract

**Background:**

Healthcare-associated infections (HAIs) pose significant challenges in skilled nursing facilities (SNFs), where residents are vulnerable due to age, comorbidities, and frequent healthcare interventions, yet infection prevention resources may be limited. To address this issue, Wayne State University partnered with Michigan Department of Health and Human Services to develop a baseline assessment with Project First Line, a CDC-based Infection Prevention and Control (IPC) training program, for staff of SNFs in Detroit.

**Objective:**

To assess baseline IPC knowledge, attitudes, and practices (KAP) of healthcare personnel (HCP) in Detroit SNFs and identify gaps to inform implementation strategies.

**Figure:**
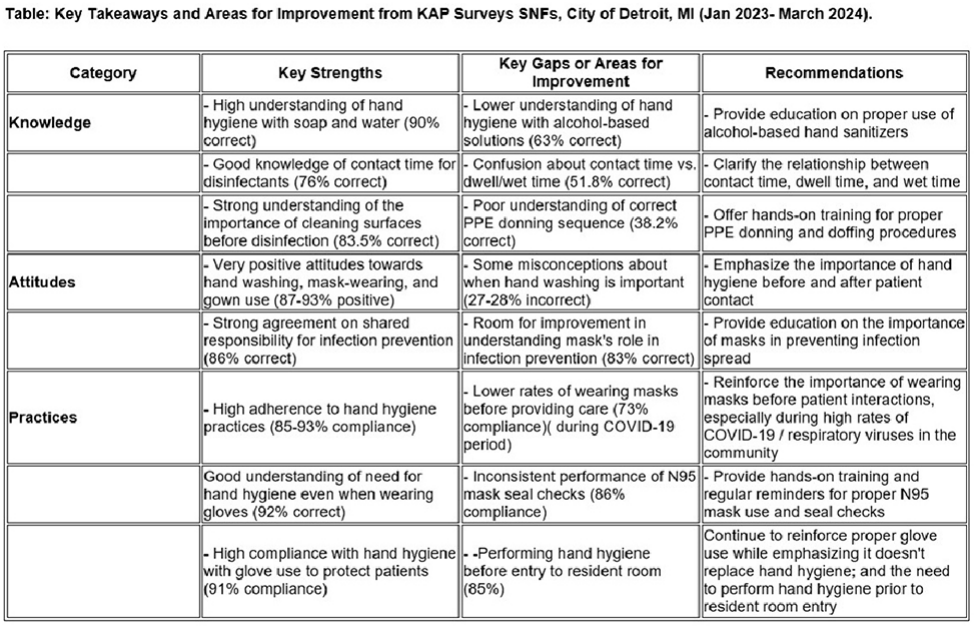

**Methods:**

A cross-sectional survey with 23 items was administered to HCPs in participating Detroit SNFs from January 2023-May 2024. The survey evaluated understanding of key IPC concepts including hand hygiene, PPE use, and environmental cleaning. Collected anonymized responses were analyzed using descriptive statistics. The project was approved by the WSU Institutional Review Board.

**Results:**

168 HCP completed the survey in eight SNFs. Participants were mostly female (82%) and Black/African American (74%), including nurses (23%), CNAs (18%), housekeeping (16%), and administrative staff (12%). Knowledge assessment revealed strong understanding of handwashing (90% correct) but lower recognition of hand sanitizer duration (63%), disinfectant contact time (76%), and isolation PPE use (71%). Despite positive attitudes toward IPC (87-93%), practice gaps included inconsistent mask use before patient care (73%) and N95 seal checks (86%). KAP scores were similar across all job categories.

**Conclusion:**

Findings highlight areas requiring focused education, particularly around alcohol-based sanitizer use and PPE protocols. The gap between positive attitudes and actual practices suggests a need for behavior-focused strategies beyond knowledge-based education. Multi-modal approaches with hands-on training, visual cues, and peer champions may prove more effective. Future research should evaluate post-intervention KAP changes and correlation with HAI rates.

**Disclosures:**

Trini A. Mathew, MD MPH FACP FIDSA, Alphabet Inc: Stocks/Bonds (Public Company)|GE Healthcare tech: Stocks/Bonds (Public Company) Paul E. Kilgore, M.D., M.P.H., GSK, Inc.: Grant/Research Support|IgY Lifesciences: Advisor/Consultant|IgY Lifesciences: Grant/Research Support|Moderna, Inc.: Grant/Research Support|Pfizer, Inc.: Grant/Research Support

